# Nevirapine induced mitochondrial dysfunction in HepG2 cells

**DOI:** 10.1038/s41598-017-09321-y

**Published:** 2017-08-23

**Authors:** Atchara Paemanee, Wannapa Sornjai, Suthathip Kittisenachai, Naraporn Sirinonthanawech, Sittiruk Roytrakul, Jeerang Wongtrakul, Duncan R. Smith

**Affiliations:** 1 Institute of Molecular Biosciences, Mahidol University, Bangkok, Thailand; 2 Genome Technology Research Unit, National Center for Genetic Engineering and Biotechnology, National Science and Technology Development Agency, Pathumthani, Thailand; 3 Research Institute for Health Sciences, Chiang Mai University, Chiang Mai, Thailand

## Abstract

Nevirapine (NVP) is a non-nucleoside reverse transcriptase inhibitor frequently used in combination with other antiretroviral agents for highly active antiretroviral therapy (HAART) of patients infected with the human immunodeficiency virus type 1 (HIV-1). However NVP can cause serious, life-threatening complications. Hepatotoxicity is one of the most severe adverse effects, particularly in HIV patients with chronic hepatitis C virus co-infection as these patients can develop liver toxicity after a relatively short course of treatment. However, the mechanism of NVP-associated hepatotoxicity remains unclear. This study sought to investigate the effect of NVP on protein expression in liver cells using a proteomic approach. HepG2 cells were treated or not treated with NVP and proteins were subsequently resolved by two-dimensional gel electrophoresis. A total of 33 differentially regulated proteins were identified, of which nearly 40% (13/33) were mitochondrial proteins. While no obvious differences were observed between NVP treated and untreated cells after staining mitochondria with mitotracker, RT-PCR expression analysis of three mitochondrially encoded genes showed all were significantly up-regulated in NVP treated cells. Mitochondrial dysfunction was observed in response to treatment even with slightly sub-optimal therapeutic treatment concentrations of NVP. This study shows that NVP induces mitochondrial dysregulation in HepG2 cells.

## Introduction

Nevirapine (NVP) is an alkyldiarylamine, containing two aryl and one alkyl groups attached to an amino group^1^ that is commonly used for the treatment of patients with acquired immunodeficiency syndrome (AIDS) and in the prevention of mother to child transmission of human immunodeficiency virus 1 (HIV-1) in resource poor countries^2, 3^. NVP is a non-nucleoside reverse transcriptase inhibitor with anti-HIV-1 reverse transcriptase activity, while HIV-2 reverse transcriptase is not inhibited by this drug^4^. The reverse transcriptase of HIV-1 functions to transcribe the viral HIV single-stranded RNA genome to a double-stranded (ds) complementary DNA as a prerequisite to integration of the viral genome in the host genome^5^. NVP can bind to the reverse transcriptase and block RNA dependent or DNA dependent polymerase activity by distorting the dNTP binding pocket^6^. Because of the development of resistance to reverse transcriptase inhibitors during HIV infection^7^ NVP is often used in combination with other antiretroviral drug^8^, although common side effects can include skin rash and nausea^9^. One of the most significant adverse effects of NVP is liver toxicity that can lead to hepatitis and death^10^. NVP associated hepatotoxicity can present as an early or late consequence of NVP treatment. The early form can occur within 6 weeks of NVP administration and is associated with skin rash and hypersensitivity and is probably immune mediated^11^, while late onset hepatotoxicity commonly starts 4–5 months after the onset of NVP treatment^10^. The mechanism of late onset hepatotoxicity of NVP remains unclear, although evidence suggests that the NVP metabolites may play a role in both the induction of the skin rash and hepatotoxicity^12^. However, in an earlier study we showed that NVP was able to induce apoptosis in HepG2 cells^13^. Significantly, HepG2 cells have low levels of phase I and phase II enzymes^14, 15^ allowing investigation into the effects of NVP on liver cells, rather than the effect of metabolites of NVP. In this study the effects of NVP treatment of HepG2 cells was further investigated through the application of proteomic analysis of proteins differentially expressed as a consequence of NVP treatment of liver cells.

## Results

### Differential expression of liver proteins after exposure to NVP

**Figure 1:**
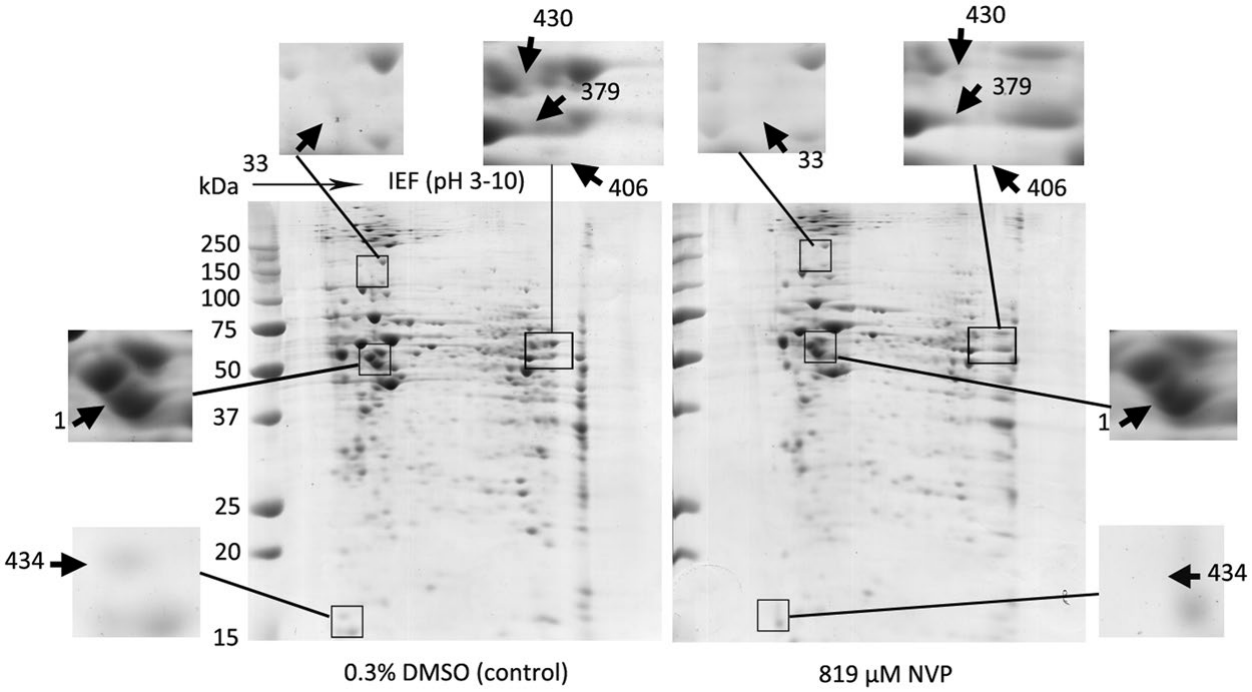
Analysis of proteins differentially expressed between control and NVP treated HepG2 cells. HepG2 cells were treated with either 819 μM NVP or 0.3% DMSO for 7 days before lysates were prepared and proteins subjected to 2D-PAGE analysis. Experiment was undertaken independently in triplicate and representative examples are shown. Selected spots are highlighted and numbering of spots corresponds to Table.

In a previous study we determined that the IC_50_ of NVP in HepG2 cells was approximately 819 μM^13^, somewhat lower than the value determined by others^16^. Long term culture of cells with 819 μM NVP results in caspase 9 mediated cell death by week 3 of culture^13^. To determine proteins whose expression is modulated by NVP, HepG2 cells were therefore cultured with 819 μM NVP for one week in parallel with cells treated with 0.3% DMSO as a vehicle control. After cell culture, proteins were isolated and resolved by 2D-PAGE (Fig. 1). Experiment was undertaken as three independent biological replicates. After scanning and spot analysis, a total of 44 significantly (p < 0.05) differentially expressed protein spots were identified. These were excised from the gels and subjected to in-gel trypic digestion and mass spectroscopic analysis of the resultant peptides. A total of 33 proteins were identified after a Mascot search^17^. Of these protein spots, seven proteins were up-regulated and 26 were down-regulated in the NVP-treated cells as compared to vehicle treated control cells. The proteins are listed in the Table 1.

**Table 1:** Proteins with altered expression in HepG2 cells after Nevirapine treatment.

**Spot No**.	**NCBInr ID (Uniprot)**	**Name**	**Mascot Score**	**MW**	**Change (fold)**
385	gi|66346721 (PCK1)	Phosphoenolpyruvate carboxykinase (GTP)	128	71451	Down/12.24
136	gi|809185 (ANXA5)	Annexin AV	302	35840	Up/2.07
208	gi|2809324 (CALU)	Calumenin	200	37164	Up/2.29
133	gi|4588526 (CLIC1)	Chloride intracellular channel protein 1	215	27249	Down/10.9
379	gi|31645 (GAPDH)	Glyceraldehyde-3-phosphate dehydrogenase	660	36201	Down/2.19
143	gi|704416 (TUFM)	Elongation factor Tu, mitochondrial	522	49852	Down/2.10
1	gi|32189394 (ATP5B)	ATP synthase subunit beta, mitochondrial	848	56525	Up/2.00
166	gi|21040386 (HSPA9)	Stress-70 protein, mitochondrial	493	73920	Down/1.52
390	gi|119600342 (ALDOA)	Fructose-bisphosphate aldolase A	111	39851	Down/15.12
434	gi|42476281 (VDAC)	Voltage-dependent anion-selective channel protein	527	32060	Down/11.97
43	gi|5803013 (ERP29)	Endoplasmic reticulum resident protein 29	202	29032	Up/3.12
217	gi|7305503 (STOML2)	Stomatin-like protein 2, mitochondrial	376	38624	Up/1.74
392	gi|4505763 (PGK1)	Phosphoglycerate kinase 1	125	44985	Down/4.63
302	gi|4758484 (GSTO1)	Glutathione S-transferase omega-1	280	27833	Up/3.54
33	gi|62203298 (IDH1)	Isocitrate dehydrogenase [NADP] cytoplasmic	479	46915	Up/2.60
412	gi|119611102 (OLFML2B)	Olfactomedin-like protein 2B	19	77239	Down/ND
404	gi|4557237 (ACAT1)	Acetyl-CoA acetyltransferase, mitochondrial	26	45456	Down/ND
406	gi|19880695 (ACTRT1)	Actin-related protein T1	21	42258	Down/ND
428	gi|4504713 (INSM1)	Insulinoma-associated protein 1	24	53916	Down/ND
418	gi|4557888 (KRT18)	Keratin, type I cytoskeletal 18	94	48029	Down/ND
315	gi|20150581 (TDP1)	Tyrosyl-DNA Phosphodiesterase 1	39	52298	Down/ND
399	gi|119631909 (NEB)	Nebulin	33	780576	Down/ND
410	gi|119606009 (HS3ST6)	Heparan sulfate (glucosamine) 3-O-sulfotransferase 6	37	56731	Down/ND
397	gi|4758988 (RAB15)	Ras-related protein Rab-15	75	24660	Down/ND
424	gi|4757810 (ATP5A1)	ATP synthase subunit alpha, mitochondrial	465	59828	Down/ND
401	gi|595266 (HADHA)	Trifunctional enzyme subunit alpha, mitochondrial	221	83688	Down/ND
383	gi|21618652 (ABAT)	4-aminobutyrate aminotransferase, mitochondrial	135	57087	Down/ND
111	gi|31815 (GLUD1)	Glutamate dehydrogenase 1, mitochondrial	56	61701	Down/ND
400	gi|37267 (TKT)	Transketolase	194	68519	Down/ND
407	gi|50592988 (UQCRC2)	Cytochrome b-c1 complex subunit 2, mitochondrial	98	48584	Down/ND
387	gi|4505621 (PEBP1)	Phosphatidylethanolamine-binding protein 1	125	21158	Down/ND
395	gi|5453543 (AKR1C1)	Aldo-keto reductase family 1 member C1	228	37221	Down/ND
430	gi|4505591 (PRDX1)	Peroxiredoxin-1	148	22324	Down/ND

ND: Not determined.

### Verification of proteomic analysis

**Figure 2:**
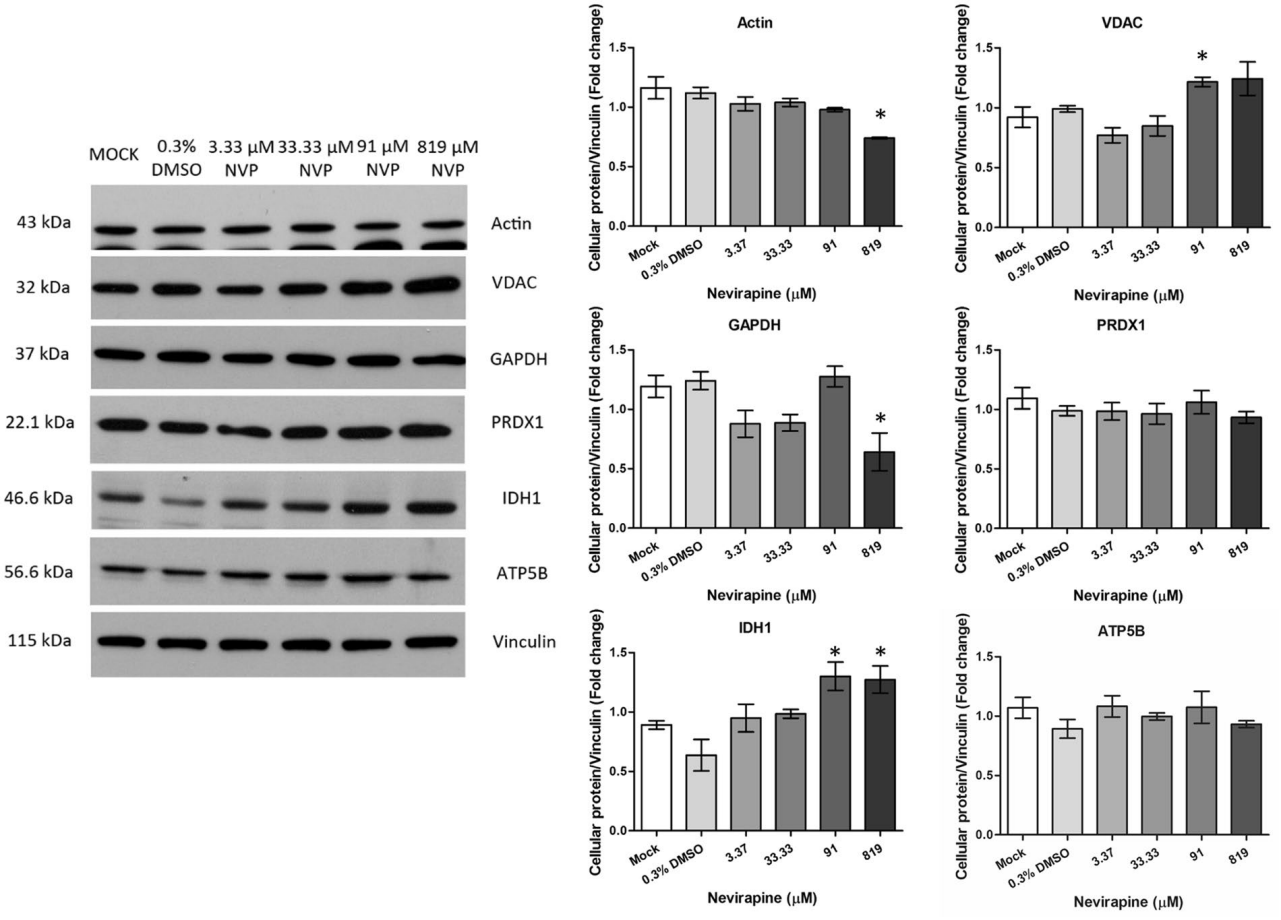
Western blot analysis of selected proteins. HepG2 cells were not treated (mock), treated with 0.3% DMSO or treated with NVP at concentrations ranging from 3.37 μM to 819 μM. On day 7 of treatment cells lysates were prepared which were subjected to SDS-PAGE and western blot analysis to detect the expression of actin, voltage dependent anion channel (VDAC), Glyceraldehyde-3-phosphate dehydrogenase (GAPDH), peroxiredoxin-1 (PRDX1), Isocitrate dehydrogenase [NADP] cytoplasmic (IDH1), ATP synthase subunit beta, mitochondrial (ATP5B) and vinculin. The experiment was undertaken independently in triplicate. Protein band intensities were quantitated using the imageJ image analysis program and analyzed by GraphPad Prism 5 program and the expression all proteins was normalized to vinculin. Error bars show S.E.M.

To validate the results of the 2D analysis, five proteins were selected for analysis, namely VDAC, GAPDH, PRDX1, IDH1 and ATP5B. Actin was originally intended as a loading control, but as early evidence suggested that this protein was additionally differentially expressed, a second loading control was used, namely vinculin and all proteins were subsequently normalized to this protein. HepG2 cells were therefore treated with NVP at final concentrations ranging from 3.37 to 819 μM in parallel with cells treated with 0.3% DMSO as a vehicle control and untreated cells (mock) as an additional control. Results (Fig. 2) confirmed the proteomic analysis for two proteins, with GAPDH being down regulated at 819 μM and IDH1 being up regulated at 91 and 819 μM. While slight reductions were seen at 819 μM for PRDX1 and ATP5B, the reductions were not statistically significant. Actin was additionally shown to be down-regulated in response to NVP treatment. VDAC was discordant in that while the 2D analysis showed the spot identified as down-regulated, Western analysis showed it was significantly up-regulated.

**Figure 3:**
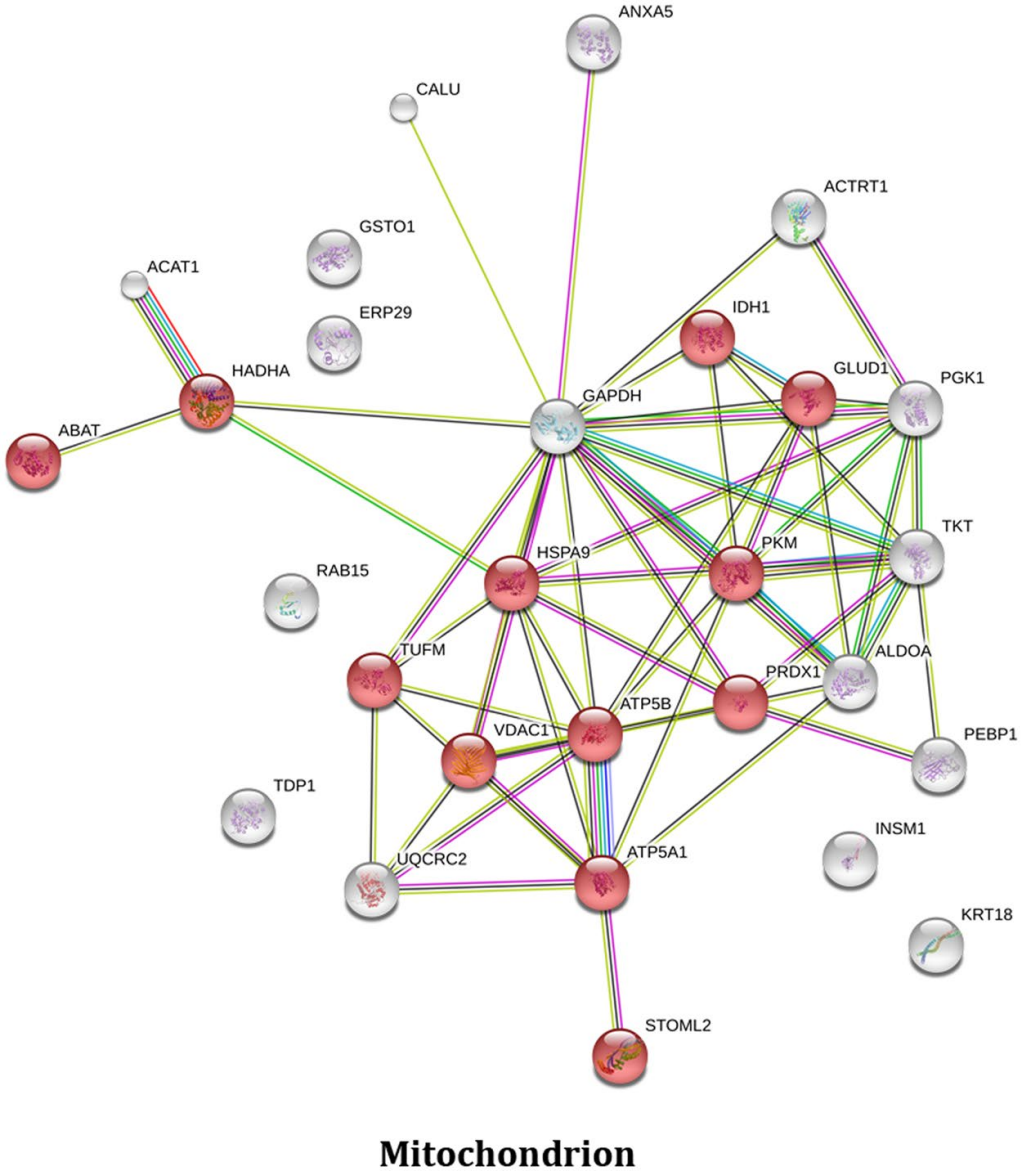
Analysis of proteins differentially expressed in NVP treated HepG2 cells. STRING analysis of proteins differentially expressed in response to NVP treatment. Not all proteins identified were mapped as some Uniprot identifiers were not recognized by SwissProt.

Bioinformatic analysis using the DAVID Bioinformatics resource^18, 19^ identified 15 functional annotation clusters of which annotation cluster 1 was associated with mitochondria (Enrichment score 3.22), while annotation cluster 2 was associated with glycolysis/gluconeogenesis (enrichment score 2.47) and annotation cluster 3 was associated with oxidation reduction (enrichment score 2.43) as shown in Supplemental File S1. A second analysis using the Gene Ontology Consortium web resource (http://geneontology.org/page/go-enrichment-analysis) based on the PANTHER classification system^20, 21^ also highlighted mitochondria (4.73 fold enrichment; P value: 5.58E-04) but additionally identified mitochondrial nucleoid proteins as being significantly over-represented in the data set (64.73 fold enrichment; P value: 2.12E-05) as shown in Supplemental File S2. In total 13 of the 33 or nearly 40% of the proteins shown to be differentially regulated are defined as mitochondrial proteins. STRING analysis^22^ identified 12 of the proteins as being associated with mitochondria (Fig. 3).

### Analysis of mitochondria after NVP treatment

**Figure 4:**
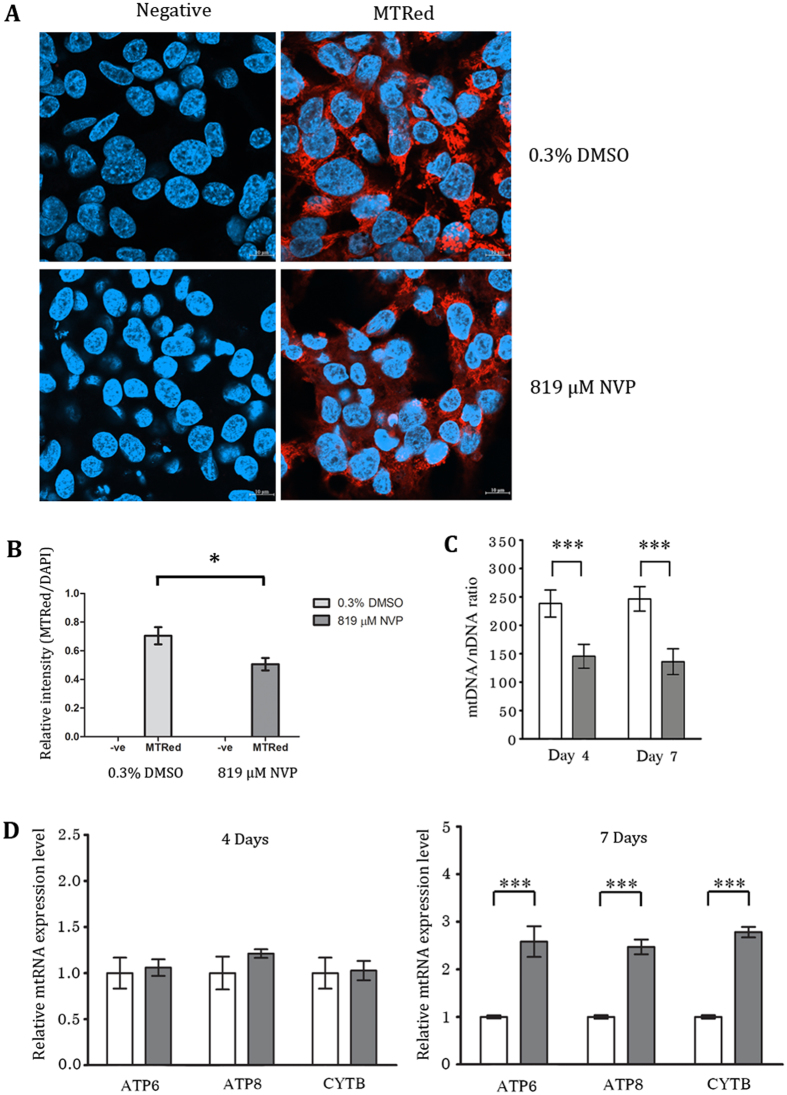
NVP treatment induced changes in mitochondria. HepG2 cells were treated with either 819 μM NVP (gray bars) or 0.3% DMSO (white bars) for 7 days (**A**,**B**) or 4 and 7 days (**C**,**D**) after which cells were (**A**) stained or not stained with mitotracker (red) and stained with DAPI (blue) and examined by confocal microscopy at x63 times magnification. Representative merged images are shown. (**B**) The mitotracker red signal was normalized against DAPI for 11 (mitotracker red treated) or 3 (no mitotracker red staining) fields and relative intensity plotted. Error bars represent S.E.M. (*p value < 0.05). (**C**) The relative mitochondrial genome number was assessed by qPCR. Mitochondrial CYTB gene (mtDNA) was normalized against nuclear FPN1A gene (nDNA). (**D**) The expression of ATP6, ATP8, CYTB and β-tubulin was assessed by RT-qPCR. The relative mitochondrial RNA expression level was normalized against control. Error bars represent SD (***p value < 0.001).

To investigate the involvement of mitochondria in NVP induced toxicity we initially treated cells with 819 μM NVP for one week and then stained the cells with mitotracker red. Cells were subsequently examined under a confocal microscope. Results (Fig. 4A) showed no obvious differences between NVP treated and untreated cells. However, quantitation of the signal intensity from the confocal imaging and normalization with the DAPI intensity showed a significant reduction in the mitotracker signal in NVP treated cells (Fig. 4B).

We therefore sought to assess the transcriptional activity of mitochondrially encoded genes. Cells were therefore again treated with NVP, and on days 4 and 7 post treatment, the expression of three mitochondrially encoded genes (ATP6, ATP8 and CYTB) was examined by qRT-PCR. Results (Fig. 4D) showed that while no difference in expression levels were seen between treated and untreated cells on day 4 of culture, the expression of all three genes was significantly higher in treated cells on day 7 of culture with NVP. We additionally quantitated the levels of mitochondria on the same days and under the same treatment condition. Levels of mitochondria were assessed by qPCR of the mitochondrial genome encoded cytochrome B (CYTB) gene with normalization against the single copy nuclear DNA encoded ferroportin 1 A gene. Results (Fig. 4C) showed a clear deficit in mitochondria in the NVP treated cells on both days examined.

### Treatment of HepG2 with sub-therapeutic levels of NVP

**Figure 5:**
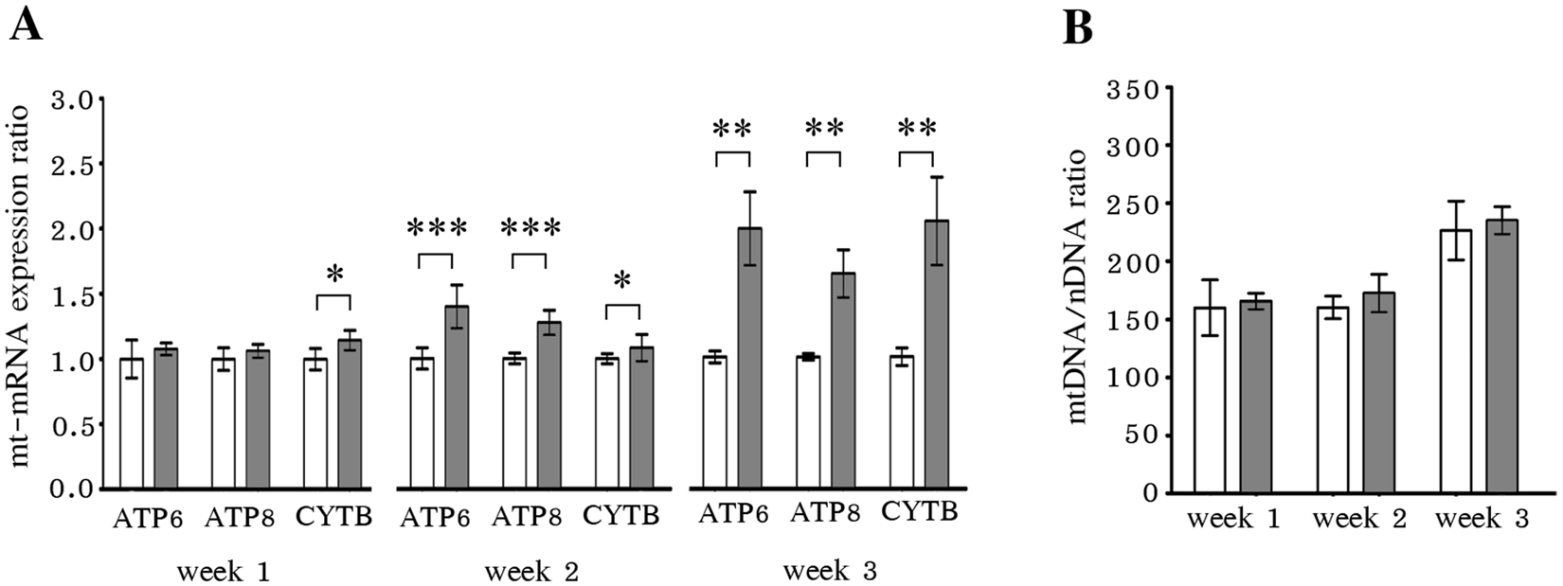
Low concentration NVP treatment induced changes in mitochondria. HepG2 cells were treated with either 10 μM NVP (gray bars) or 0.0037% DMSO (white bars) for 1, 2 or 3 weeks after which cells were (**A**) examined for the expression of ATP6, ATP8, CYTB and β-tubulin by RT-qPCR with the expression of the mitochondrial RNA being normalized against β-tubulin and the relative expression level was calculated using 2^−∆∆CT^ method, and (**B**) the relative mitochondrial genome number was assessed by qPCR with the mitochondrial CYTB gene (mtDNA) being normalized against nuclear FPN1A gene (nDNA). Error bars represent SD (*p value < 0.05, **p value < 0.01 and ***p value < 0.001).

**Figure 6:**
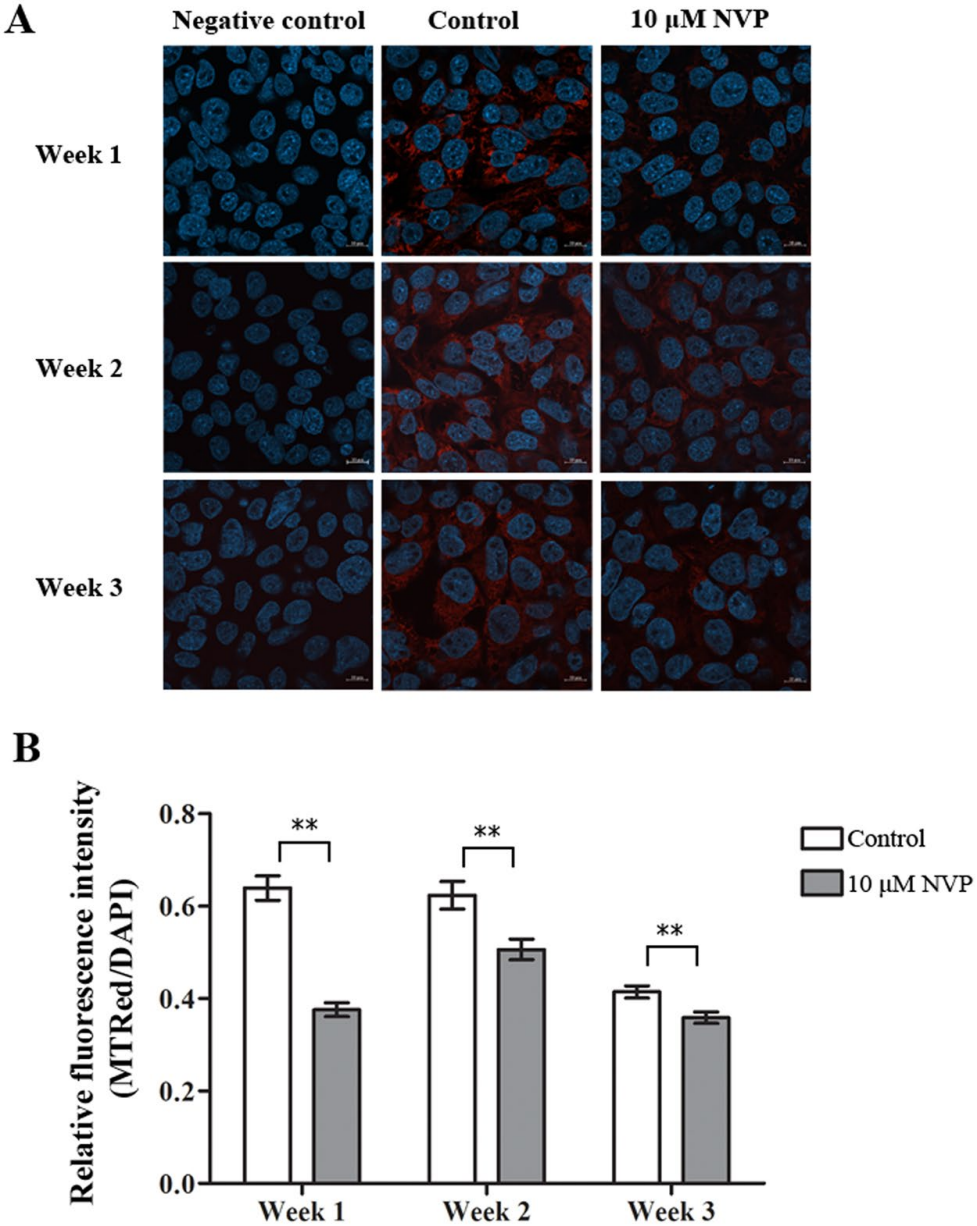
Low concentration NVP treatment induced changes in mitochondria as assessed by mitotracker staining. HepG2 cells were treated with either 10 μM NVP (gray bars) or 0.0037% DMSO (white bars) for 1, 2 or 3 weeks after which cells were (**A**) stained or not stained with mitotracker (red) and stained with DAPI (blue) and examined by confocal microscopy at x63 times magnification. Representative merged images are shown. (**B**) The mitotracker red signal was normalized against DAPI for 25 (mitotracker red treated) or 10 (no mitotracker red staining) fields and relative intensity plotted. Error bars represent S.E.M. (**p value < 0.01).

To determine the effects of NVP on mitochondria under more physiologically relevant conditions, a lower concentration was used. The recommended therapeutic serum concentration for NVP is 3400–8000 ng/ml^23^ corresponding to 12 to 30 μM, and so cells were cultured without NVP or with NVP at 10 μM (slightly below the lower limit therapeutic level) for up to three weeks. After weeks 1, 2 and 3 the levels of expression of the mitocondrially encoded ATP6, ATP8 and CYTB was determined by qRT-PCR and the relative levels of mitochondria were determined by qPCR. In addition the cells were again examined by confocal microscopy to determine the relative intensity of mitotracker. Consistent with the results at higher levels of NVP treatment there was dysregulation of expression of mitochondrial genes (Fig. 5A). Increased CYTB expression was observed from week 1, while all genes examined showed increased expression by weeks 2 and 3. At the lower level of treatment no deficit in mitochondria number was seen as assessed by qPCR (Fig. 5B), although and increase mtDMA/nDNA was observed in week 3, but this possibly relates to minor variation in culture conditions as importantly, the mtDMA/nDNA ration was increased equally for both NVP treated and non-treated cells. However, again a significantly lower intensity of mitotracker signal was observed at all times points tested (Fig. 6). These results again support the observation that NVP treatment can affect mitochondrial function.

## Discussion

The bioactivation of NVP by cytochrome P450 proteins is proposed to be the main mechanism of NVP induced hepatotoxicity in patients treated with this drug^12^. This bioactivation occurs as part of the detoxification process which is divided into three phases. Phase I enzymes such as cytochrome P450 proteins mediate the initial detoxification process^24^ generating hydroxyl-metabolites such as 2-OH-NVP, 3-OH-NVP and 12-OH-NVP^25^. 12-OH-NVP can be O-sulfonated to the protein reactive 12-Sulfoxyl-NVP^25^. However, other pathways involving the generation of NVP-epoxides have been proposed^26, 27^ with phase II enzymes such as glutathione S-transferase mediating the subsequent glutathionylation of the metabolite^24^. Phase III enzymes mediate the subsequent excretion of the metabolites from the cells. HepG2 cells have been characterized as having low expression of phase I and II enzymes^14, 15^ and are thus a suitable model system to dissect out any effects of the parental drug, as differentiated from the metabolites of this drug.

Treatment of HepG2 cells with 819 μM NVP induces apoptosis within three weeks^13^. To understand this process, HepG2 cells were treated with 819 μM NVP for one week in parallel with vehicle treated cells and differential protein expression determined by 2D-PAGE. A total of 44 spots were identified, which lead to the identification of 33 differentially expressed proteins. Western blot validation of the proteome analysis showed two proteins whose differential expression agreed with the 2D-PAGE analysis, while two further showed slight, but non-significant reductions in expression in response to NVP treatment.

Most interestingly, nearly 40% of these proteins were identified as mitochondrial proteins. While the complete mitochondrial proteome has yet to be fully determined, studies suggest that as many as 1500 proteins may comprise the mitochondrial proteome^28^. While mitochondria have their own genetic material, this only encodes for some 13 polypeptides which are critical proteins in the process of oxidative phosphorylation^29^, and the vast majority of proteins associated with mitochondria are encoded in the nuclear genome.

VDAC was one of the mitochondrial proteins identified as differentially regulated in this study, although while the 2D-analysis indicated that this protein was down-regulated, western blot analysis showed significant up-regulation. However, there are three known cellular isoforms of VDAC, VDAC1 to VDAC3^30^. These proteins are present at different abundances within the cell, and VDAC1 is ten times more abundant than VDAC2, which in turn is 10 times more abundant than VDAC3^31^. It is possible therefore that the spot detected as down-regulated represented one of the less abundant isoforms of VDAC. While VDAC is present in the plasma membrane^32^, VDAC primarily functions as a conductance controlled pore in the outer mitochondrial membrane, controlling the release of adenine nucleotides^33^, calcium ions^34^ and other metabolites of the cell^35^.

The increase in expression of VDAC as determined by western blot analysis is mirrored by an increase in transcription of the mitochondrially encoded ATP synthase F0 subunits 6 and 8 (ATP6 and ATP8) and mitochondrial cytochrome b (CYTB). These results collectively suggest increased activity of mitochondria in response to NVP treatment, although it was clear that short term treatment (4 days) did not affect gene transcription. While no apparent gross morphological changes were observed in mitochondria after mitotracker staining, a reduction of signal intensity was observed upon analysis, suggesting loss of mitochondria. This would suggest that the increased transcription observed in the mitochondrially encoded genes is a compensatory mechanism for the loss of mitochondria.

While the identification of the dysfunction of mitochondria was undertaken with concentrations of NVP outside of the plasma therapeutic range of 3400–8000 ng/ml^23^, analysis of mitochondria under a slightly sub-therapeutic range concentration of NVP confirmed that NVP treatment resulted in mitochondrial dysfunction in HepG2 cells. Under both high and low NVP treatment expression of genes encoded by the mitochondrial genome was elevated, and mitotracker intensity was reduced. Loss of mitochondria was observed under the high NVP treatment regime, but not low NVP treatment suggesting that a longer period of treatment at the lower concentration of NVP may be required to recapitulate the loss of mitochondria.

The loss of mitochondria during anti-HIV therapy has been well documented^36^. The loss is closely associated with treatment with nucleoside reverse transcriptase inhibitors (NRTIs) which are believed to inhibit a number of mitochondrial enzymes including DNA polymerase gamma^37, 38^, the enzyme responsible for mitochondrial DNA replication^39^. NRTIs have also been associated with the reduction in expression and activity of mitochondrially encoded members of the respiratory chain^40, 41^. Few studies have linked NNRTIs to mitochondrial dysfunction, although efavirenz has been shown to induce mitochondrially mediated apoptosis in Jurkat cells as well as in primary T cells of uninfected donors^42^ and to induce mitochondrial membrane depolarization of lymphocytes of HIV-1 infected HAART patients^43^. Similarly, mitochondrially mediated apoptosis has been implicated as a consequence of NVP treatment in lymphocytes of HIV-1 infected HAART patients^43^ and in liver (HepG2) cells^13^. Other studies in liver cells (Hep3B) have suggested that while efavirenz can induce mitochondrial dysfunction, similar results were not observed with NVP treatment^44^. However, there are distinct differences in the abundance of expression of drug-metabolizing genes between HepG2 and Hep3B^15^ that may explain the discrepancy between the study of Blas-Garcia and colleagues^44^ and the results seen here.

Overall, this study has shown that there is distinct mitochondrial dysfunction in HepG2 cells treated with NVP. How the mitochondrial dysfunction links to the induction of apoptosis of liver cells treated with NVP^13^ will require further investigation, as will determining the potential clinical significance of these observations in liver cells with normal levels of phase I and phase II enzymes.

## Materials and Methods

### Cell culture

The human hepatocellular carcinoma cell line HepG2 (ATCC-Number HB-8065) was maintained in Dulbecco’s modified Eagle’s medium (DMEM) supplemented with 10% heat inactivated fetal bovine serum (FBS), 100 U/ml penicillin and 100 µg/ml streptomycin in a tissue culture flask at 37 °C in 5% CO_2_. Where indicated cells were treated with NVP (Sigma-Aldrich Corp., Saint Louis, MO) which was prepared as a 0.273 M stock solution in DMSO.

### Protein extraction and quantitation

Cell pellets (1.9 × 10^6^ cells) were resuspended in RIPA-PIC buffer (100 mM Sodium phosphate, 150 mM Sodium chloride, 0.1% dodium dodecyl sulfate, 0.5% sodium deoxycholate and 1x protease inhibitor cocktail) and chilled on ice. The cell suspensions were mixed by vortexing and subsequently sonicated for 10 min after which solutions were centrifuged at 13,000 rpm 15 min and the supernatant was stored at −80 °C until required. Protein concentrations were determined using the Bradford assay. For 2D PAGE, proteins were precipitated from the supernatant by the addition of methanol/acetone in the ratio of 1:2:6 (sample:methanol:acetone) and dried. Precipitates were dissolved in lysis C buffer containing 8 M urea, 2 M Thiourea, 4% CHAPS, 50 mM dithiothreitol, 1 mM PMSF (Phynylmethylsulphonyl fluoride) and 1 mM Benzamidine and protein concentrations were again determined using the Bradford assay. Samples were stored at −80 °C prior to electrophoresis.

### Two dimensional electrophoresis

Two-dimensional gel electrophoresis was performed essentially as described elsewhere^45, 46^. Briefly, a total of 165 μg of each sample were loaded onto Immobiline Drystrip (pH 3–10 NL, 7 cm) gels in a final volume of 130.5 μl containing 2% IPG buffer (Amersham BioSciences UK Ltd., Little Chalfont, Buckinghamshire, UK) and 0.004% bromophenol blue and strips rehydrated for 12 h. Proteins were subjected to isoelectric focusing using a Ettan IPGphore II system (Amersham BioSciences) at 20 °C (300 volts for 200 Vhr, 1,000 volts for 300 Vhr, 3,000 volts for 4,000 Vhr, 5,000 volts for 4,500 Vhr and 5,000 volts to reach 3,000 volt-h). The maximum current was maintained at 50μA per IPG strip. Strips were subsequently reduced in equilibration buffer (50 mM Tris pH 8.8, 6 M urea, 30% glycerol, 2% SDS, and 0.002% bromophenol blue) supplemented with 57 mM DTT for 15 min and alkylated in equilibration buffer with 135 mM iodoacetamide. Second dimension electrophoresis was undertaken on a 12.5% polyacrylamide gel run at an applied voltage of 100 volts until the bromophenol blue dye front reached 0.5 cm from the bottom of the gel. Gels were subsequently stained with 0.25% Coomassie Brilliant Blue R250 in a solution containing 50% methanol, 10% glacial acetic acid for 48 h and destained in a solution with 16.5% ethanol, 5% glacial acetic acid for 6 h. Stained 2D gels were scanned under visible light at 300 μm/pixel resolution. Image data were analyzed using Image Master 2D Platinum software version 7.0 (GeneBio, Geneva Bioinformatics, Geneva, Switzerland) and spots were evaluated on intensity, volume and area. Statistical analysis was performed by one-way analysis of variance, with a p value of 0.05 being considered significant.

### Tryptic in-gel digestion and LC MS/MS

Spots identified as differentially regulated were removed from the gel and subjected to in-gel digestion as modified from Shevchenko and colleagues [8]. Briefly, gel plugs were dehydrated with 100% acetonitrile (ACN) and carbamidomethylation was performed by reducing with 10 mM DTT in 10 mM ammonium bicarbonate at 56 °C for 1 hr and alkylating with 100 mM iodoacetamide in 10 mM ammonium bicarbonate for 1 hr. Subsequently the gel pieces were dehydrated with 100% ACN for 5 min. In-gel digestion of proteins was performed using 10 ng/μl trypsin in 10 mM ammonium bicarbonate and incubation at 37 °C for 3 hr. Tryptic peptides were extracted using 30 μl of 50% ACN in 0.1% formic acid. Mass of peptides was determined by LC-MS/MS using a SYNAPT HDMS (Waters Corp., Milford, MA). The Nanoscale LC system was equipped with a Symmetry C18 5 µm, 180-μm × 20-mm Trap column and a BEH130 C18 1.7 µm, 100-µm × 100-mm analytical reversed phase column (Waters Corp., Milford, MA) and samples were eluted at a flow rate of 600 nL/min under gradient conditions of 15–50% B over 15 min. The mobile phase A consisted of water with 0.1% formic acid and mobile phase B consists of acetonitrile with 0.1% formic acid. Mass spectral data from 300 to 1800 m/z was collected in the positive ionization mode. The MS/MS spectrometry data were searched against human protein database from NCBInr and Swissprot using the MASCOT search engine^17^.

### Western blot analysis

HepG2 cells cultured under standard conditions without or without NVP as required were washed with ice cold PBS and lysed with RIPA buffer (1% Nonidet P-40, 0.5% sodium deoxycholate, 0.1% SDS in PBS). The cell lysates were incubated on ice for 30 min with periodic mixing then sonicated twice at 4 °C for 5 min before centrifugation at 10,000 × g for 10 min and supernatants were collected. Proteins were quantified by the Bradford method (Bradford, 1976) and kept at −80 °C until use. A total of 30 μg of total proteins were separated by electrophoresis through a 12% SDS polyacrylamide gel and transferred to a nitrocellulose membrane by electroblotting. The membranes were blocked with 5% skim milk in TBST (20 mM Tris, 140 mM NaCl, and 0.1% Tween-20) at room temperature for 1 h with shaking. The membranes were probed overnight at 4 °C with a 1:3,000 dilution of a goat polyclonal anti actin antibody (sc-1616; Santa Cruz Biotechnology, Inc., Santa Cruz, CA), or a 1:1,000 dilution of a mouse monoclonal anti-glyceraldehyde 3-phosphate dehydrogenase (GAPDH) antibody (sc-32233; Santa Cruz Biotechnology, Inc.), or a 1:3,000 dilution of a rabbit monoclonal anti-voltage dependent anion channel (VDAC) antibody (4661; Cell Signaling, Danvers, MA), or a 1:50,000 dilution of a goat polyclonal anti- peroxiredoxin 1 (PRDX1) antibody (EB09018; One World Lab, San Diego CA), or a 1:1,000 dilution of a rabbit polyclonal anti- isocitrate dehydrogenase 1 (IDH1) antibody (AP7454c; One World Lab), or a 1:2,000 dilution of a mouse monoclonal anti-mitochondrial ATP synthase subunit beta (ATP5B) antibody (TA500850; One World Lab), or a 1:5,000 dilution of a goat polyclonal anti-vinculin antibody (sc-7649; Santa Cruz Biotechnology, Inc).

After washing with TBST three times for 5 min each time, the membranes were incubated as appropriate with a 1:5,000 dilution of a horseradish peroxidase (HRP)-conjugated polyclonal rabbit anti-goat polyclonal antibody (31402; Pierce, Rockford, IL), a 1:5,000 (for GAPDH) or a 1:10,000 (for PRDX1) dilution of a HRP-conjugated polyclonal goat anti-mouse IgG antibody (A4416; Sigma-Aldrich), or a 1:10,000 dilution of a HRP-conjugated polyclonal goat anti-rabbit IgG (31460; Pierce) in TBST at room temperature for 1 h, and specific protein bands were visualized with Clearity^TM^ ECL Western Blotting Substrate (Bio-Rad Laboratories, Inc., Hercules, CA). Signals were recorded by film autoradiography.

### Mitotracker fluorescent staining

HepG2 cells were seeded in 6-well plates containing coverslips at a density of 1.0 × 10^6^ cells/well. Cells were grown for 20 hours then treated with 819 μM of NVP for up to 7 days and 0.3% DMSO treated cells were used as a control. Treatment of cells with 10 μM of NVP was performed for up to 3 weeks with replacement of NVP solution every 4 days and 0.0037% DMSO treated cells were used as a control. Both NVP treated HepG2 cells and control were collected and washed in 1xPBS (137 mM NaCl_2_, 2.7 mM KCl, 4.3 mM Na_2_HPO_4_, 1.47 mM KH_2_PO_4_). For MitoTracker® Red CMXRos staining (Molecular Probes, Eugene, OR), cells were submerged in 500 nM of MitoTracker staining solution and incubated in 5% CO_2_ incubator at 37 °C for 15 minutes. After staining, cells were gently washed in 1x PBS for four times. After washing, HepG2 cells were fixed by 3.7% formaldehyde for 20 minutes. After fixation, the cells were washed twice in 1x PBS buffer for 3 minutes at room temperature. After washing, the fixed cells were permeablilized with 0.3% Triton-X in PBS-IFA (0.5 M Na_2_HPO_4_, 0.5 M KH_2_PO_4_, 1.5 M NaCl_2_) in the dark for 10 min. After incubation, cells were washed twice in 0.3% Triton-X 100. The permeablilized cells were submerged in cold acetone for 5 minutes at room temperature and washed in 0.03% Triton-X in PBS-IFA for 5 minutes for 4 times. Nuclear staining of the cells was performed using a 1:50 dilution of DAPI (Merck Millipore, Temecula, CA) as counterstaining. After 1 hour incubation in the dark, cells were washed in 0.03% Triton-X in 1x PBS-IFA 5 minutes for 4 times. For mounting the coverslips, Prolong® Gold artifade (Invitrogen, Eugene, Oregon, USA) was dropped onto a glass slide and coverslips were placed upside down on Prolong® overnight at 4 °C in dark.

### Confocal imaging

MitoTracker Red fluorescence was acquired with a Carl Zeiss Laser Scanning Confocal Microscope (LSM 800) using 578 nm excitation and 598 emission filter setting. DAPI fluorescence was acquired using 353 nm excitation and 465 emission filter setting. Detector gains were set for MitoTracker Red and DAPI at 700 and 650 V respectively. Imaging analysis was performed using ZEN software version 2.1. Intensity of mitotracker and DAPI signals was collected over 11 (819 μM NVP treatment) or 25 (10 μM NVP treatment) image fields for NVP treated and non-treated cells (3 fields for negative, non-mitotracker stained cells) and the mitotracker signal was normalized to the DAPI signal.

### RNA extraction

HepG2 cells were treated with 819 μM of NVP for 4 and 7 days and 0.3% DMSO treated cells were used as a control. Both control and NVP treated HepG2 cells were collected and total RNA was isolated with TRIzol reagent (Life Technologies Inc., Carlsbad, CA) according to the manufacturer’s instructions. RNA concentration was measured by a Nanodrop-2000 (Thermo Fisher Scientific Inc., Wilmington, DE). The RNA purity was verified by using the absorbance ratio at OD 260/280 for which all samples were in range of a 1.8–2.0 ratio. Contaminating genomic DNA was removed by DNase I (Life Technologies Inc.) treatment. After digestion, DNase I enzyme was removed by re-extraction with TRIzol reagent.

### RT-qPCR

The extracted RNA was used as a template for cDNA synthesis by using RevertAid Reverse Transcriptase (Thermo Fisher Scientific Inc., Waltham, MA). The reverse transcription PCR was undertaken using a VeritiTM Thermo Cycler (Applied Biosystems, Foster City, CA) and the reaction containing with 1 μg total RNA, 1 μM random hexamer, 1X reaction buffer and 200 U RevertAid Reverse Transcriptase. After cDNA synthesis, the expression of three mitochondrial genes ATP6, ATP8 and CYTB was determined by quantitatative PCR and β-tubulin was used as an internal control. The amplification was performed using KAPA SYBR FAST qPCR Kit 2X Master MIX (Kapa Biosystems Inc., Woburn, MA.) and the following specific primers: ATP6-f 5′-CTCTATTGATCCCCACCTCC-3′, ATP6-r 5′-TGGTAAGAAGTGGGCTAGGG-3′, ATP8-f 5′-ATGCCCCAACTAAATACTACCG-3′, ATP8-r 5′-TGGGGGCAATGAATGAAGCG-3′, CYTB-f 5′-GCCCTCGGCTTACTTCTCTT-3′, CYTB-r 5′-AGTGATTGGCTTAGTGGGCG-3′, β-tubulin-f 5′CTGGCACCATGGACTCTG3′ and β-tubulin-r 5′TCGGCTCCCTCTGTGTAG3′. The reactions were carried out in a Mastercycler realplex (Eppendorf AG, Hauppauge, NY) using the following conditions: initial denaturation at 95 °C for 3 min and then denaturation at 95 °C for 10 sec, annealing at 60 °C for 30 sec and extension at 72 °C for 20 sec for 40 cycles. The expression of mitochondrial RNA was normalized against β-tubulin and the relative expression level was calculated using 2^−∆∆CT^ method (∆∆CT = ∆CT_treat_ − ∆CT_control_, ∆CT = Ct_target_ − Ct_β-tubulin_).

### DNA extraction and quantitation of mitochondria

Control and NVP treated HepG2 cells were collected at the appropriate time points and DNA was isolated with TRIzol reagent (Life Technologies Inc., Carlsbad, CA) according to the manufacturer’s protocol. DNA concentrations were measured using a Nanodrop-2000 (Thermo Fisher Scientific Inc., Wilmington, DE). DNA purity was verified by determining the absorbance ratio of OD 260/280 and all samples were in the ratio range 1.8–2.0. Mitochondria were quantitated by qPCR essentially as described previously^47^, with each reaction containing 50 ng DNA template, 1X KAPA SYBR FastMaster Mix (Kapa biosystems, Inc., Wilmington, MA.) and 300 nM of the following specific primers: CYTB-mtDNA-f 5′AACTTCGGCTCACTCCTTGG3′, CYTB-mtDNA-r 5′CCAATGTATGGGATGGCGGA3′, FPN1A-nDNA-f 5′-CAAACCGCTTCCATAAGGCTTTGC3′, FPN1A-nDNA-r 5′-TTCTGCGGCTGCTATCGCTG-3′. The amplification reactions were carried out in a Mastercycler realplex (Eppendorf AG, Hauppauge, NY) with the following conditions: initial denaturation at 95 °C for 3 min and then denaturation at 95 °C for 10 sec, annealing at 60 °C for 30 sec and extension at 72 °C for 20 sec for 40 cycles. The relative mitochondrial DNA copy number was normalized against FPN1A gene.

### Statistical analysis

All data were analyzed using the GraphPad Prism program (GrapPad Software Inc., San Diego, CA). Statistical analysis of significance was undertaken by One-Way ANOVA with or without LSD Post Hoc multiple comparisons on raw data reads using SPSS (SPSS Inc., Chicago, IL). Real time PCR data was evaluated by independent sample *t*-tests. Data was considered as a statistical significant at a *p* value of less than 0.05.

### Data availability statement

All data generated or analysed during this study are included in this published article (and its Supplementary Information files).

## Electronic supplementary material

Supplementary information
