# Supplementary material for: Nevirapine induced mitochondrial dysfunction in HepG2 cells
Source: Sci Rep. 2017 Aug 23;7:9194. doi: 10.1038/s41598-017-09321-y (PMC5569014; doi:10.1038/s41598-017-09321-y)
Supplement: Supplementary file 1 — Supplementary information [file 41598_2017_9321_MOESM1_ESM.pdf]

Supplementary Files

Nevirapine induced mitochondrial dysfunction in HepG2 cells

Atchara Paemanee[a,b], Wannapa Sornjai[a], Suthathip Kittisenachai[b], Naraporn Sirinonthanawech[a], Sittiruk Roytrakul[b], Jeerang Wongtrakul[c*], Duncan R. Smith[a, *]

[a]Institute of Molecular Biosciences, Mahidol University, Bangkok, Thailand

[b]Genome Technology Research Unit, National Center for Genetic Engineering and Biotechnology, National Science and Technology Development Agency, Pathumthani, Thailand

[c]Research Institute for Health Sciences, Chiang Mai University, Chiang Mai, Thailand

* Address correspondence to either:

Duncan R. Smith, duncan.smi@mahidol.ac.th; duncan_r_smith@hotmail.com

or

Jeerang Wongtrakul, jeerang@gmail.com

Supplemental File S1: Full list of annotation clusters identified by DAVID

Supplemental File S2: Full list of cellular components identified by PANTHER

Functional Annotation Clustering

Help and Manual

Current Gene List: List_2
Current Background: Homo sapiens
33 DAVID IDs

Options **Classification Stringency** Medium

Rerun using options | Create Sublist

15 Cluster(s)

Download File

Annotation Cluster 1	Enrichment Score: 3.22	G		Count	P_Value	Benjamini
GOTERM_CC_FAT	mitochondrial part	RT		12	1.3E-8	1.5E-6
GOTERM_CC_FAT	mitochondrial matrix	RT		9	1.5E-8	8.3E-7
GOTERM_CC_FAT	mitochondrial lumen	RT		9	1.5E-8	8.3E-7
GOTERM_CC_FAT	nucleoid	RT		5	4.8E-7	1.8E-5
GOTERM_CC_FAT	mitochondrial nucleoid	RT		5	4.8E-7	1.8E-5
GOTERM_CC_FAT	mitochondrion	RT		13	7.0E-7	2.0E-5
UP_SEQ_FEATURE	transit peptide:Mitochondrion	RT		9	7.5E-7	2.4E-4
SP_PIR_KEYWORDS	transit peptide	RT		9	8.3E-7	6.2E-5
SP_PIR_KEYWORDS	mitochondrion	RT		10	5.8E-6	2.2E-4
GOTERM_CC_FAT	mitochondrial membrane	RT		8	1.3E-5	3.0E-4
GOTERM_CC_FAT	mitochondrial envelope	RT		8	1.9E-5	3.7E-4
GOTERM_CC_FAT	organelle envelope	RT		9	2.9E-5	4.7E-4
GOTERM_CC_FAT	envelope	RT		9	2.9E-5	4.2E-4
GOTERM_CC_FAT	mitochondrial inner membrane	RT		7	3.5E-5	4.4E-4
GOTERM_CC_FAT	organelle inner membrane	RT		7	5.2E-5	5.9E-4
GOTERM_CC_FAT	intracellular organelle lumen	RT		12	5.6E-4	5.8E-3
GOTERM_CC_FAT	organelle lumen	RT		12	6.8E-4	6.5E-3
GOTERM_CC_FAT	membrane-enclosed lumen	RT		12	8.1E-4	7.1E-3
GOTERM_CC_FAT	organelle membrane	RT		8	6.4E-3	4.8E-2
KEGG_PATHWAY	Parkinson's disease	RT		4	8.2E-3	7.1E-2
KEGG_PATHWAY	Alzheimer's disease	RT		4	1.6E-2	9.1E-2
GOTERM_BP_FAT	oxidative phosphorylation	RT		3	1.6E-2	4.2E-1
KEGG_PATHWAY	Huntington's disease	RT		4	2.1E-2	1.0E-1
GOTERM_CC_FAT	mitochondrial membrane part	RT		3	2.8E-2	1.7E-1
SP_PIR_KEYWORDS	mitochondrion inner membrane	RT		3	4.1E-2	3.1E-1
KEGG_PATHWAY	Oxidative phosphorylation	RT		3	6.9E-2	2.7E-1
SP_PIR_KEYWORDS	ion transport	RT		4	7.0E-2	4.4E-1
SP_PIR_KEYWORDS	transport	RT		6	1.4E-1	6.6E-1
GOTERM_BP_FAT	ion transport	RT		4	2.0E-1	9.0E-1
GOTERM_BP_FAT	phosphorylation	RT		4	2.1E-1	9.0E-1
SP_PIR_KEYWORDS	hydrolase	RT		5	2.6E-1	8.7E-1
GOTERM_BP_FAT	phosphorus metabolic process	RT		4	3.1E-1	9.5E-1
GOTERM_BP_FAT	phosphate metabolic process	RT		4	3.1E-1	9.5E-1
GOTERM_BP_FAT	transmembrane transport	RT		3	3.2E-1	9.5E-1
SP_PIR_KEYWORDS	membrane	RT		10	7.6E-1	1.0E0
GOTERM_CC_FAT	plasma membrane	RT		6	9.4E-1	1.0E0
**Annotation Cluster 2**	**Enrichment Score: 2.47**	**G**		**Count**	**P_Value**	**Benjamini**
SP_PIR_KEYWORDS	gluconeogenesis	RT		4	4.0E-6	2.0E-4
KEGG_PATHWAY	Glycolysis / Gluconeogenesis	RT		4	9.4E-4	3.3E-2
SP_PIR_KEYWORDS	glycolysis	RT		3	2.4E-3	4.4E-2
GOTERM_BP_FAT	glucose metabolic process	RT		4	3.4E-3	4.3E-1
GOTERM_BP_FAT	glycolysis	RT		3	3.9E-3	3.9E-1
GOTERM_BP_FAT	glucose catabolic process	RT		3	5.9E-3	4.5E-1
GOTERM_BP_FAT	hexose metabolic process	RT		4	6.4E-3	4.1E-1
GOTERM_BP_FAT	hexose catabolic process	RT		3	8.3E-3	4.5E-1
GOTERM_BP_FAT	monosaccharide catabolic process	RT		3	8.8E-3	4.2E-1

Category	Term		Count	P_Value	Benjamini
Annotation Cluster 1	Enrichment Score: 3.22		Count	P_Value	Benjamini
GOTERM_BP_FAT	monosaccharide metabolic process	RT	4	9.5E-3	4.1E-1
GOTERM_BP_FAT	alcohol catabolic process	RT	3	1.1E-2	4.3E-1
GOTERM_BP_FAT	cellular carbohydrate catabolic process	RT	3	1.2E-2	4.3E-1
GOTERM_BP_FAT	carbohydrate catabolic process	RT	3	2.0E-2	4.1E-1
Annotation Cluster 3	Enrichment Score: 2.43		Count	P_Value	Benjamini
GOTERM_MF_FAT	NAD or NADH binding	RT	4	1.5E-4	2.8E-2
SP_PIR_KEYWORDS	oxidoreductase	RT	7	2.9E-4	8.5E-3
GOTERM_BP_FAT	oxidation reduction	RT	7	1.4E-3	2.9E-1
GOTERM_MF_FAT	cofactor binding	RT	5	2.2E-3	9.4E-2
GOTERM_MF_FAT	coenzyme binding	RT	4	7.5E-3	1.8E-1
SP_PIR_KEYWORDS	nadp	RT	3	2.8E-2	2.4E-1
INTERPRO	NAD(P)-binding domain	RT	3	3.3E-2	8.3E-1
SP_PIR_KEYWORDS	nad	RT	3	3.9E-2	3.1E-1
Annotation Cluster 4	Enrichment Score: 2.2		Count	P_Value	Benjamini
KEGG_PATHWAY	Propanoate metabolism	RT	3	4.9E-3	8.5E-2
KEGG_PATHWAY	Butanoate metabolism	RT	3	5.5E-3	6.5E-2
KEGG_PATHWAY	Valine, leucine and isoleucine degradation	RT	3	9.2E-3	6.4E-2
Annotation Cluster 5	Enrichment Score: 1.67		Count	P_Value	Benjamini
GOTERM_CC_FAT	intracellular non-membrane-bounded organelle	RT	12	1.2E-2	8.4E-2
GOTERM_CC_FAT	non-membrane-bounded organelle	RT	12	1.2E-2	8.4E-2
GOTERM_CC_FAT	cytoskeleton	RT	7	6.4E-2	3.0E-1
Annotation Cluster 6	Enrichment Score: 1.57		Count	P_Value	Benjamini
GOTERM_BP_FAT	ATP biosynthetic process	RT	3	1.4E-2	4.1E-1
GOTERM_BP_FAT	purine ribonucleoside triphosphate biosynthetic process	RT	3	1.6E-2	4.2E-1
GOTERM_BP_FAT	purine nucleoside triphosphate biosynthetic process	RT	3	1.7E-2	4.1E-1
GOTERM_BP_FAT	ribonucleoside triphosphate biosynthetic process	RT	3	1.7E-2	4.1E-1
GOTERM_BP_FAT	nucleoside triphosphate biosynthetic process	RT	3	1.7E-2	4.0E-1
GOTERM_BP_FAT	ATP metabolic process	RT	3	1.8E-2	4.0E-1
GOTERM_BP_FAT	purine ribonucleotide biosynthetic process	RT	3	2.3E-2	4.3E-1
GOTERM_BP_FAT	purine ribonucleoside triphosphate metabolic process	RT	3	2.3E-2	4.3E-1
GOTERM_BP_FAT	ribonucleoside triphosphate metabolic process	RT	3	2.3E-2	4.2E-1
GOTERM_BP_FAT	purine nucleoside triphosphate metabolic process	RT	3	2.4E-2	4.2E-1
GOTERM_BP_FAT	ribonucleotide biosynthetic process	RT	3	2.5E-2	4.1E-1
GOTERM_BP_FAT	nitrogen compound biosynthetic process	RT	4	2.6E-2	4.1E-1
GOTERM_BP_FAT	nucleoside triphosphate metabolic process	RT	3	2.8E-2	4.2E-1
GOTERM_BP_FAT	purine ribonucleotide metabolic process	RT	3	3.1E-2	4.4E-1
GOTERM_BP_FAT	ribonucleotide metabolic process	RT	3	3.4E-2	4.5E-1
GOTERM_BP_FAT	purine nucleotide biosynthetic process	RT	3	3.5E-2	4.5E-1
GOTERM_BP_FAT	purine nucleotide metabolic process	RT	3	5.3E-2	5.6E-1
GOTERM_BP_FAT	nucleotide biosynthetic process	RT	3	5.3E-2	5.6E-1
GOTERM_BP_FAT	nucleobase, nucleoside, nucleotide and nucleic acid biosynthetic process	RT	3	5.6E-2	5.7E-1
GOTERM_BP_FAT	nucleobase, nucleoside and nucleotide biosynthetic process	RT	3	5.6E-2	5.7E-1
Annotation Cluster 7	Enrichment Score: 1.38		Count	P_Value	Benjamini
GOTERM_CC_FAT	pigment granule	RT	4	8.5E-4	6.9E-3
GOTERM_CC_FAT	melanosome	RT	4	8.5E-4	6.9E-3
GOTERM_CC_FAT	membrane-bounded vesicle	RT	5	3.0E-2	1.7E-1
GOTERM_CC_FAT	vesicle	RT	5	5.0E-2	2.6E-1
GOTERM_CC_FAT	cytoplasmic membrane-bounded vesicle	RT	4	1.1E-1	4.5E-1
GOTERM_CC_FAT	cytoplasmic vesicle	RT	4	1.5E-1	5.6E-1
GOTERM_CC_FAT	endoplasmic reticulum	RT	3	6.1E-1	9.7E-1
GOTERM_CC_FAT	extracellular region	RT	4	8.2E-1	1.0E0
Annotation Cluster 8	Enrichment Score: 1.17		Count	P_Value	Benjamini
SP_PIR_KEYWORDS	P-loop	RT	4	9.3E-4	2.3E-2
SP_PIR_KEYWORDS	nucleotide-binding	RT	9	5.3E-3	7.7E-2
GOTERM_MF_FAT	nucleotide binding	RT	12	6.6E-3	1.8E-1
SP_PIR_KEYWORDS	gtp-binding	RT	4	1.7E-2	1.7E-1
GOTERM_MF_FAT	purine ribonucleotide binding	RT	9	4.3E-2	6.4E-1
GOTERM_MF_FAT	ribonucleotide binding	RT	9	4.3E-2	6.4E-1

	Category	Term			Count	P_Value	Benjamini
	Annotation Cluster 1	Enrichment Score: 3.22	G		Count	P_Value	Benjamini
☐	GOTERM_MF_FAT	GTP binding	RT		4	4.9E-2	6.4E-1
☐	GOTERM_MF_FAT	guanyl ribonucleotide binding	RT		4	5.3E-2	6.3E-1
☐	GOTERM_MF_FAT	guanyl nucleotide binding	RT		4	5.3E-2	6.3E-1
☐	GOTERM_MF_FAT	purine nucleotide binding	RT		9	5.4E-2	6.1E-1
☐	SP_PIR_KEYWORDS	atp-binding	RT		6	6.6E-2	4.3E-1
☐	UP_SEQ_FEATURE	nucleotide phosphate-binding region:GTP	RT		3	8.7E-2	1.0E0
☐	GOTERM_MF_FAT	purine nucleoside binding	RT		7	1.4E-1	8.8E-1
☐	GOTERM_MF_FAT	nucleoside binding	RT		7	1.4E-1	8.7E-1
☐	GOTERM_CC_FAT	cell surface	RT		3	1.7E-1	5.8E-1
☐	GOTERM_BP_FAT	phosphorylation	RT		4	2.1E-1	9.0E-1
☐	GOTERM_MF_FAT	ATP binding	RT		6	2.3E-1	9.6E-1
☐	GOTERM_MF_FAT	adenyl ribonucleotide binding	RT		6	2.4E-1	9.6E-1
☐	GOTERM_MF_FAT	adenyl nucleotide binding	RT		6	2.7E-1	9.7E-1
☐	GOTERM_BP_FAT	phosphorus metabolic process	RT		4	3.1E-1	9.5E-1
☐	GOTERM_BP_FAT	phosphate metabolic process	RT		4	3.1E-1	9.5E-1
☐	UP_SEQ_FEATURE	nucleotide phosphate-binding region:ATP	RT		3	4.8E-1	1.0E0
	Annotation Cluster 9	Enrichment Score: 0.93	G		Count	P_Value	Benjamini
☐	GOTERM_BP_FAT	response to organic substance	RT		6	1.3E-2	4.2E-1
☐	GOTERM_BP_FAT	response to organic cyclic substance	RT		3	2.4E-2	4.2E-1
☐	GOTERM_CC_FAT	soluble fraction	RT		4	2.8E-2	1.7E-1
☐	GOTERM_BP_FAT	response to hormone stimulus	RT		3	1.7E-1	8.8E-1
☐	GOTERM_CC_FAT	cell fraction	RT		5	1.9E-1	6.2E-1
☐	GOTERM_BP_FAT	response to endogenous stimulus	RT		3	1.9E-1	9.0E-1
☐	GOTERM_CC_FAT	cytosol	RT		5	3.1E-1	8.0E-1
☐	GOTERM_CC_FAT	membrane fraction	RT		3	5.2E-1	9.5E-1
☐	GOTERM_CC_FAT	insoluble fraction	RT		3	5.4E-1	9.6E-1
	Annotation Cluster 10	Enrichment Score: 0.88	G		Count	P_Value	Benjamini
☐	GOTERM_BP_FAT	protein homooligomerization	RT		3	1.5E-2	4.2E-1
☐	GOTERM_BP_FAT	protein oligomerization	RT		3	4.7E-2	5.3E-1
☐	GOTERM_MF_FAT	lipid binding	RT		4	7.8E-2	7.1E-1
☐	GOTERM_BP_FAT	protein complex assembly	RT		3	2.7E-1	9.3E-1
☐	GOTERM_BP_FAT	protein complex biogenesis	RT		3	2.7E-1	9.3E-1
☐	GOTERM_BP_FAT	macromolecular complex assembly	RT		3	3.9E-1	9.8E-1
☐	GOTERM_BP_FAT	macromolecular complex subunit organization	RT		3	4.2E-1	9.8E-1
	Annotation Cluster 11	Enrichment Score: 0.73	G		Count	P_Value	Benjamini
☐	GOTERM_BP_FAT	negative regulation of apoptosis	RT		3	1.6E-1	8.8E-1
☐	GOTERM_BP_FAT	negative regulation of programmed cell death	RT		3	1.6E-1	8.8E-1
☐	GOTERM_BP_FAT	negative regulation of cell death	RT		3	1.6E-1	8.8E-1
☐	GOTERM_BP_FAT	regulation of apoptosis	RT		4	2.1E-1	9.0E-1
☐	GOTERM_BP_FAT	regulation of programmed cell death	RT		4	2.2E-1	9.0E-1
☐	GOTERM_BP_FAT	regulation of cell death	RT		4	2.2E-1	9.0E-1
	Annotation Cluster 12	Enrichment Score: 0.66	G		Count	P_Value	Benjamini
☐	GOTERM_BP_FAT	intracellular protein transport	RT		3	1.7E-1	8.8E-1
☐	GOTERM_BP_FAT	protein transport	RT		4	1.9E-1	9.1E-1
☐	GOTERM_BP_FAT	establishment of protein localization	RT		4	2.0E-1	9.0E-1
☐	GOTERM_BP_FAT	cellular protein localization	RT		3	2.0E-1	8.9E-1
☐	GOTERM_BP_FAT	cellular macromolecule localization	RT		3	2.0E-1	8.9E-1
☐	GOTERM_BP_FAT	protein localization	RT		4	2.6E-1	9.3E-1
☐	GOTERM_BP_FAT	intracellular transport	RT		3	3.8E-1	9.8E-1
	Annotation Cluster 13	Enrichment Score: 0.6	G		Count	P_Value	Benjamini
☐	GOTERM_BP_FAT	synaptic transmission	RT		3	1.2E-1	8.1E-1
☐	GOTERM_BP_FAT	transmission of nerve impulse	RT		3	1.5E-1	8.8E-1
☐	GOTERM_BP_FAT	behavior	RT		3	2.4E-1	9.1E-1
☐	GOTERM_BP_FAT	cell-cell signaling	RT		3	3.4E-1	9.6E-1
☐	GOTERM_BP_FAT	neurological system process	RT		3	7.1E-1	1.0E0
	Annotation Cluster 14	Enrichment Score: 0.17	G		Count	P_Value	Benjamini
☐	GOTERM_MF_FAT	calcium ion binding	RT		4	3.4E-1	9.9E-1
☐	GOTERM_MF_FAT	ion binding	RT		10	6.4E-1	1.0E0
☐	SP_PIR_KEYWORDS	metal-binding	RT		5	7.5E-1	1.0E0
☐	GOTERM_MF_FAT	metal ion binding	RT		9	7.5E-1	1.0E0

	Annotation Cluster 1	Enrichment Score: 3.22	G			Count	P_Value	Benjamini
☐	GOTERM_MF_FAT	cation binding	RT			9	7.6E-1	1.0E0
☐	GOTERM_MF_FAT	transition metal ion binding	RT			4	9.6E-1	1.0E0
	Annotation Cluster 15	Enrichment Score: 0.03	G			Count	P_Value	Benjamini
☐	SP_PIR_KEYWORDS	cell membrane	RT			4	7.2E-1	1.0E0
☐	GOTERM_CC_FAT	plasma membrane	RT			6	9.4E-1	1.0E0
☐	UP_SEQ_FEATURE	transmembrane region	RT			4	9.9E-1	1.0E0
☐	SP_PIR_KEYWORDS	transmembrane	RT			4	9.9E-1	1.0E0
☐	GOTERM_CC_FAT	integral to membrane	RT			4	1.0E0	1.0E0
☐	GOTERM_CC_FAT	intrinsic to membrane	RT			4	1.0E0	1.0E0

42 terms were not clustered.

Analysis Summary: Please report in publication

**Analysis Type:** PANTHER Overrepresentation Test (release 20160715)		
**Annotation Version and Release Date:** GO Ontology database Released 2016-11-30		
**Analyzed List:**	upload_1 (Homo sapiens)	Change
**Reference List:**	Homo sapiens (all genes in database)	Change
**Annotation Data Set:** GO cellular component complete		
☑ Use the Bonferroni correction for multiple testing		

Results

	Reference list	upload_1
Mapped IDs:	20972 out of 20972	33 out of 36
Unmapped IDs:	0	0
Multiple mapping information:	0	3

Bonferroni count: 1304

Export results

Displaying only results with P<0.05; click here to display all results

	Homo sapiens (REF)	upload_1 (Hierarchy NEW!)				
GO cellular component complete	#	#	expected	Fold Enrichment	+/-	P value
mitochondrial nucleoid	45	5	.08	64.73	+	2.12E-05
↳organelle	13117	35	22.52	1.55	+	1.35E-03
↳mitochondrion	1725	14	2.96	4.73	+	5.58E-04
↳cytoplasmic part	8168	30	14.02	2.14	+	7.74E-05
↳cytoplasm	10829	32	18.59	1.72	+	3.13E-03
↳membrane-bounded organelle	12169	34	20.89	1.63	+	1.44E-03
↳nucleoid	47	5	.08	61.97	+	2.62E-05
↳mitochondrial part	1002	11	1.72	6.40	+	7.61E-04
↳mitochondrial matrix	428	9	.73	12.25	+	4.58E-05
myelin sheath	179	9	.31	29.29	+	2.40E-08
mitochondrial inner membrane	505	8	.87	9.23	+	2.44E-03
↳mitochondrial membrane	700	8	1.20	6.66	+	2.63E-02
↳mitochondrial envelope	741	8	1.27	6.29	+	3.94E-02
↳organelle envelope	1155	11	1.98	5.55	+	3.05E-03
↳envelope	1161	11	1.99	5.52	+	3.21E-03
↳organelle inner membrane	563	8	.97	8.28	+	5.43E-03

extracellular matrix	522	8	.90	8.93	+	3.11E-03
↳extracellular region part	3841	28	6.59	4.25	+	1.90E-11
↳extracellular region	4564	29	7.83	3.70	+	1.30E-10
adherens junction	684	8	1.17	6.81	+	2.23E-02
↳anchoring junction	702	8	1.21	6.64	+	2.68E-02
extracellular exosome	2750	26	4.72	5.51	+	9.88E-13
↳extracellular vesicle	2764	26	4.74	5.48	+	1.12E-12
↳vesicle	4051	27	6.95	3.88	+	1.01E-09
↳extracellular organelle	2765	26	4.75	5.48	+	1.13E-12
